# Application of Dance Movement Therapy to Life-Death Education of College Students Under Educational Psychology

**DOI:** 10.3389/fpsyg.2022.782771

**Published:** 2022-04-05

**Authors:** Liu Yang, Fen Li

**Affiliations:** ^1^College of Arts, Hunan University of Arts and Science, Changde, China; ^2^Faculty of Music and Performing Arts, Sultan Idris Education University, Tanjung Malim, Malaysia; ^3^Management College, Shinawatra University, Bangkok, Thailand

**Keywords:** educational psychology, college students’ life education, dance movement therapy, body awareness, deep learning

## Abstract

The present work aims to efficiently carry out life-death education (LDE) for college students, improve their psychological problems, and reduce suicide accidents by combining LDE with Dance Movement Therapy (DMT). DMT is a psychosomatic cross therapy that treats mental or physical diseases through dance or improvisation. Firstly, this paper introduces LDE and DMT and designs the activities of DMT intervention. Secondly, the relationship between DMT and LDE is analyzed. Finally, a questionnaire survey is conducted on the research objects. The research objects are divided into the experimental group receiving DMT intervention, the control group participating in sports dance courses, and the benchmark group. The research data show no significant difference in interpersonal skills and emotional psychology among the three groups. The DMT intervention group has substantial changes in other factors except for the conflict control ability before and after the intervention. In addition, the questionnaire survey results after the intervention demonstrate that the DMT intervention group gets a significantly higher score in the interpersonal relationship, interpersonal relationship building ability, moderate rejection ability, self-disclosure ability, and emotional support ability. Therefore, DMT intervention positively impacts college students’ interpersonal relationships. After the sports dance course, there are differences in the total score of interpersonal ties and scores of interpersonal relationships building ability, self-disclosure ability, and emotional support ability in the control group, but with no significant difference in moderate rejection ability and conflict control ability. Therefore, compared with traditional psychotherapy methods, the DMT method reported here is conducive to releasing pressure and alleviating physical and mental anxiety. The research content provides new ideas for psychological education in colleges and universities and contributes to improving college students’ suicidal tendencies and helping college students grow up healthily.

## Introduction

Entering college from high school is a crucial turning point in students’ life. College students face a series of problems, such as study, emotion, interpersonal relationships, and life planning. In the crucial period of psychological growth, college students need to receive appropriate psychological counseling to establish and improve their mental health and avoid potential psychological problems. Unfortunately, college students’ psychological problems emerge one after another in real life. For example, some college students only pay attention to professional ability and ignore other necessary skills. Some are excessively addicted to beer and skittles, and some lack interpersonal skills. Consequently, these students may suffer from various psychological problems. In recent years, some college students who have entered colleges and universities have finally been suspended, dropped out, or even committed suicide due to psychological problems. This situation is undoubtedly heart-wrenching. Dance Movement Therapy (DMT) is a psychosomatic cross therapy to treat mental and physical diseases through dance or improvisation. Dance can strengthen the body and make people with mental illnesses happy by conveying ideas and expressing emotions. DMT is a new therapeutic method different from traditional therapy. Physically, it helps patients keep healthy and make patients like sports. Psychologically, it can help patients obtain happiness, enhance self-confidence, improve temperament, and get rid of the shackles of psychological obstacles in essence. Therefore, DMT is of great significance for college students’ mental health and can guide them to understand themselves correctly. The present work studies the intervention effect of DMT on college students’ interpersonal relationships and emotional and mental health development. In recent years, the annual statistics from the *National Suicide Prevention and Control Center* have shown a dramatic increase in the suicide rate of Chinese college students. The probable cause is that many college students are helpless without a positive outlook under even more fierce social pressure, difficulties in employment, and setbacks in a unique growth environment. Some even make irreparable mistakes (Liu et al., 2019). Furthermore, the ascending suicide rate suggests that college students lack proper life cognition and Life-Death Education (LDE) (Li et al., 2020). LDE, also called death education, can help people understand that life and death are inevitable natural human life to help them establish a scientific, reasonable, and healthy concept of death. Meanwhile, it can also eliminate people’s fear and anxiety about death and let people think deeply about death with an ordinary heart and learn the psychological process of death and the psychological impact on people. In this way, people can be psychologically ready to deal with the death of themselves and their relatives. Besides, they can face the problems of life, old age, and death bravely and turn their understanding of life and death into a powerful driving force to cherish life and health and improve their quality of life. In addition, LDE can cultivate people to face death calmly and consciously improve their superior life with the concept of quality of life in the three stages of birth, survival, and death. Specifically, the purpose of the LDE course is to help learners understand the knowledge of medicine, philosophy, and sociology related to death so that people can correctly treat life, setbacks, and disasters in life (Malin et al., 2020). In particular, college years are the critical phase of college students’ psychological and physiological maturity and personality formation. Hence, school education inevitably affects them (Woodford et al., 2018). Thus, the thoughts and beliefs formed during this period will help them pursue their life objectives and determine future development. However, the current educational system in China neglects to help students cultivate essential qualities. For example, it has been reported that one in every four Chinese college students has claimed to be aimless. Meanwhile, they show increasingly strong material desire under rapid technological development and commercialization. Worse still, there is a proliferation of college students feeling confused and unbacked, which is a pressing problem worthy of primary social concern from all walks of life (Rogoza et al., 2018; Zhang et al., 2018).

As an innovative approach for college education, LDE aims to encourage students to explore and understand the meaning of life, respect and cherish lives, and develop a harmonious relationship between heaven, earth, man, and the self (Shin et al., 2018). So far, LDE has grown into an indispensable part of the higher education curriculum. In LDE, teachers are consultants rather than guides to help students analyze the meaning of life and cultivate self-esteem and positive thinking against difficulties and setbacks (Jordan, 2018). The humanistic and material environment of the campus is the main factor to promote the formation of students’ personalities and life values. They exchange ideas, learn new things, present their dynamic development and learning status, find their direction of life, and ultimately figure out the meaning of life (Wang, 2019). Briefly, there is an increasing need to pay special attention to LDE for college students. LDE can help them cultivate a healthy personality, a correct understanding of life’s significance, and a positive and challenging spirit against difficulties and setbacks to contribute to social stability and progress (Acharya et al., 2018).

To sum up, applying DMT to LDE can alleviate college students’ suicidal tendency under family, academic, and social pressure and help college students deeply understand the meaning of life. Firstly, this paper designs the activities of DMT intervention. Secondly, the connection between DMT and LDE is discussed to realize the collaborative application. Finally, a questionnaire survey is designed for three research object groups: DMT intervention experimental group, sports dance course control group, and benchmark group. The psychological status of college students is studied according to different groups and different psychological analysis factors. The main contribution of this work is to combine DMT with LDE to form mental health dance therapy, which is verified by the changes in interpersonal skills and emotional psychology. Applying DMT to college students’ LDE is conducive to college students’ introspection and emotion regulation. This scheme provides a reference for educational psychology research in the future.

## Related Works

### Life-Death Education of College Students

“LDE Center” has first seen its appearance in Sydney, Australia. Its purpose is to help people fight against drug abuse, violence, and Acquired Immune Deficiency Syndrome. Gradually, the Australian LDE Center involves all aspects of care for human life, happiness, assistance, and the value and meaning of life (Kümpfen and Maurer, 2018). Some scholars argue LDE to be the core of education since it can enrich and enhance the importance and value of human life. Initially, LDE aimed to extend human life.

There have been many arguments on the significance of LDE. For example, LDE is a kind of primary education to teach or assist students in developing correct outlooks on life, happiness, academic performance, and the culture on campus. Furthermore, LDE fosters students’ sense of life’s meaning, purpose, value, function, and limitations, encouraging them to cherish life and respect themselves, others, the environment, and nature. Padgett believed that people could learn the significance of life by themselves; someone who grew up by himself could prove his existence and succeed through his creativity (Viere et al., 2021). Temane studied college students’ opinions on the meaning of life in a university in South Africa through a questionnaire survey. They found that family expectations, education, and achievements were the most influential factors in understanding the meaning of lives (Oliynyk et al., 2020). Al-Zoubi (2020) reported that LDE indeed provided help and change to college students. Wang and Tian (2019) conducted ten counseling sessions for aggressive adolescents (once a week) to verify the impact of DMT/exercise therapy on them. These students completed a questionnaire before treatment, after treatment, and 3 months after treatment, respectively. The survey found that aggressive behavior decreased significantly after the intervention, exceptionally verbal and physical aggression and suspicion. Besides, the respondents had a better interpersonal relationship with others and higher enthusiasm for activities. These improvements remained after 3 months, proving the positive effect of DMT/exercise therapy on aggressive adolescents (Wang and Tian, 2019). Suzi and Tortora’s observed that DMT/exercise therapy based on child oncology was little known and underutilized, so they discussed DMT and pediatric tumors. Specifically, they defined medical DMT as a psychotherapy method to meet the psychosocial needs of patients and defined its difference from dance and therapeutic dance used for entertainment during hospitalization. Moreover, they provided a literature review of DMT with medically ill children in the United States and worldwide, culminating with a focus on advancements in the field, discussing the future of pediatric medical DMT (Tortora, 2019). Noticeably, many higher institutions are facing incrementing student drop-outs. The main reasons behind this are summarized below.

On the one hand, mental disorders have become the principal reason behind college drop-outs. Specifically, 6 ∼ 25.4% of college students in China have mental illnesses, such as anxiety, fear, neurasthenia, depression, or obsessive-compulsive disorder (Alexander et al., 2021; Deng et al., 2021).

On the other hand, a hierarchical pyramid structure of higher institutions is forming with the state advocational policies on higher education. Large numbers of low-performance and population-intensive universities come into being. Consequently, many college students find it difficult to find satisfactory jobs and begin to doubt the essentiality of higher education. In addition, due to the regional development gap, rural students might lack the same initiative and academic foundations for self-learning and self-improvement as urban students (Rogti, 2020). Therefore, this group is more vulnerable to learning difficulties without proper guidance, which aggravates the idea of education-uselessness and causes many students to drop out of school.

The meaning of life is an exciting and vital topic, especially for college students. LDE encourages college students to face difficulties and setbacks (Borg et al., 2021). The present work focuses on the influence of LDE on college students’ outlooks on life, way of thinking, and understanding of the meaning of life.

### Relevant Theories of Life-Death Education

Ogawa believes that LDE should train students to pay attention to their life quality and furnish them with information, understanding, skills, and strategies they require to make safe decisions about their health and well-being. LDE can teach students knowledge and technique to create their own lives with self-confidence, rationality, power, and morality (Phan et al., 2021). The goal of LDE in America is set based on four aspects: life awareness, life relationships, personal endurance, and life values. The actual contents of LDE include death education, character education, frustration education, livelihood education, and personalized education. LDE is not an independent but multidisciplinary course (Testoni et al., 2019). In Britain, LDE mainly covers two aspects. One is civic education supplemented by a health education curriculum. The other is the education of life relationships, including the relationship between one’s own life and the life of others, one’s own life and society, and one’s own life and environment. The education of life relationships teaches students to live harmoniously with others, the community, and the environment (Wyckoff and Preston, 2021).

This paper holds that the government and all sectors of society should pay attention to LDE and vigorously publicize the purpose and significance of LDE to give full play to the role of LDE. Besides, it is essential to mobilize the strength of the overall society, especially the academic circles and front-line educators. Moreover, it is imperative to conduct theoretical and practical research on LDE. In addition, LDE courses need to be designed according to the specific problems in college students’ lives and their understanding ability at different stages. Finally, it is necessary to excavate various ways to implement LDE and train many specialized teachers to improve the overall LDE system (Ekberg et al., 2019).

### Dance Movement Therapy Theory and Related Research

The meaning of dance might be enriched by associating it with treatment. As a branch of expression therapy, DMT is different from the general treatment (Michels et al., 2018). DMT can improve people’s physical health indexes, such as strength, flexibility, muscle tension, and coordination. Specifically, DMT can be used for mental rehabilitation treatment, auxiliary intervention for autistic children, stress reduction for high officials and white-collar workers, health guidance for interpersonal communication of college students, and correct guidance for technical movements of professional athletes (Wang et al., 2019). In general, dance is mostly a hobby rather than a professional sport, thus gaining wide popularity among the public (Goodill, 2021). Here, the intervention subjects of DMT are chosen from ordinary college students in the Xi’an Institute of Physical Education. The DMT intervention aims to assist students in coping with their common interpersonal and emotional problems to facilitate their healthy development (Campbell, 2019).

Professional dances require expertise and focus on skills, such as ballet, Latin dance, folk dance, and tap dance. In contrast, DMT involves more freestyle, diversified, and relaxing body movements and uses these movements as assessment tools and a primary mode of intervention. Meanwhile, DMT usually integrates such favorable elements as games, massage, speech, and painting into the intervention process (Grasser et al., 2019).

Speech (verbal communication) is vitally significant in DMT. Usually, teachers use words to guide dancers to adjust their movements or correct each other’s movements (Prieto, 2020). Verbal communication in DMT helps the therapist accurately understand and distinguish what happens in the treatment process and clearly express the intention represented by some vague and abstract body movements in the dance. Therapists can also help patients deeply understand and accept their true self or vulnerable self by words (instead of the false self or idealized self) (Tavormina and Tavormina, 2018).

The present work aims to explore the effect of DMT intervention on college students’ interpersonal communication and emotional psychology and promote college students’ mental health.

### Theory of Introspection and Dance Movement Therapy

In psychology, self refers to the feeling that an individual is aware of his existence, containing various physical and psychological characteristics, activities, and processes (Sousa et al., 2019). In particular, introspection can be used for individual self-evaluation to adjust their behaviors properly or develop toward the ideal performance. For example, the theory of introspection argues that objective introspection is a process of triggering events. In other words, individuals constantly observe themselves under particular circumstances through introspection to achieve as an ideal performance as possible.

Fox explored the influence of mood and introspection on people’s intentions and actual behaviors to help others. He found that more helpful behaviors could be triggered under a positive mindset than negative ones, and the relationship between mood and introspection was distinct (Fox et al., 2021). Biondo and Gerber (2020) suggested that DMT/exercise therapy was a promising treatment option for people with acute schizophrenia that might lead to speech obstacles, alternative reality basis, and motor dysfunction related to schizophrenia. Cramer (2018) discovered that physical and mental education courses had a significant treatment effect on shoulder and neck pains, alleviated physical and psychological pressure, and improved body awareness. To sum up, the above research implies that body awareness can be enhanced through practice and learning, and body movement education positively affects the development of body awareness.

Body awareness refers to human consciousness to perceive one’s own body, focus on own feelings, and concern subconscious behaviors. DMT affects the human body where all human awareness relies on. Therefore, body awareness can be enhanced through DMT; correspondingly, body awareness sensitivity can be improved.

### Emotion Regulation and Dance Movement Therapy

Emotion regulation is the ability of individuals to monitor, evaluate, and regulate their emotions to respond appropriately to external events. Under effective emotion management and control, an individual can interact favorably with society through awareness, expression, introspection, and emotion adjustment strategies. Meanwhile, it can explain emotional withdrawal in a non-emotional way and express emotions positively while avoiding negative expressions. It has been argued that depression is a mental response of humans, which negatively influences positive emotions.

Khan pointed out that dance was a pleasant and feasible exercise. Compared with other forms of sports, it was also influential in improving multiple health indicators. Additionally, it could become a multifunctional activity with aesthetic expression, attractive to men and women. It was also an effective tool for cultivating self-confidence, especially for the disabled. According to the *European Association for DMT*, DMT filled the gap between the advantages of dance and the difficulties of sports activities by combining expressive exercise and creativity (Khan, 2020; Ye and Chen, 2021). Dryman and Heimberg (2018) revealed that proficient emotion regulators developed fewer depressive symptoms. Patel (2020) found a negative correlation between adolescents’ emotion regulation abilities and the possibility of acquiring distress or depression. Music and body movements are the fundamental DMT elements, and their emotion-regulation effects are discussed explicitly in the present work. In earlier research, Cook observed four musical elements, i.e., rhythm, musicality, cultural shock, and relevance. The author proved that music was a crucial emotion regulation strategy and highlighted the potential use of music as a tool for emotion regulation (Cook et al., 2019; Feng et al., 2021).

Most scholars believe that individuals’ unique experiences can be well-presented through their unique behavioral expressions. Accordingly, there have been various attempts, such as art therapy, to enhance the individual’s awareness, improve their self-expression abilities, and help them regulate their emotions.

## Materials and Methods

### Application of Deep Learning to College Students’ Psychological Health

Psychology aims to describe, explain, predict, and control behaviors. To date, most psychological studies have analyzed the relationship between description and explanatory variables, and there are few studies oriented to prediction. Moreover, some traditional psychological studies have contradictory or uncertain results due to the relatively small available samples, low data quality, and lack of covariate information. In addition, the significance test based on P-value is not reproducible. By contrast, DL is a strict and systematic cross-validation technology and can improve psychological research’s replicability. DL-based technologies can implement the learning model under massive amounts of data, accurately identify the potential laws of the data, and have enhanced generalization ability, improving the model compatibility while minimizing the prediction error. For example, DL-based prediction approaches for individual complex psychological problems are on tryouts, aiming at stress, anxiety, and suicide. These methods have proven feasible through DL-based analysis of thousands of practical features. Pioneering projects, such as Microblog Social Media Suicide Recognizer, can evaluate microblog users’ suicide intention in real-time over a multi-layer perceptron algorithm, with a prediction accuracy as high as 94%. The statistical methods used here are the controlled trial and questionnaire survey. The controlled trial method divides the research objects into the experimental and control group. The experimental group receives DMT intervention, and the control group takes dance courses. The effectiveness of the DMT method proposed here is verified by comparing the experimental and control groups’ treatment results. The Interpersonal Competence Questionnaire of College Students (ICQ-CS) and the Positive and Negative Affect Scale (PANAS) are adopted to test the effect of the DMT intervention experiment. The ICQ-CS includes five dimensions: interpersonal relationship building ability, moderate rejection ability, self-disclosure ability, conflict control ability, and emotional supportability. Meanwhile, the PANAS is employed to investigate the emotion of the experimental subjects. Both scales use Likert’s five-point scoring method to collect data.

### Research Subjects

A total of 90 students from Xi’an Institute of Physical Education are randomly selected as research subjects divided into the experimental group for DMT intervention, the control group for the sports dance course, and the benchmark group (Suskin and Al-Yagon, 2020). Sports dance is designed for the requirements of physical education reform in higher institutions. It is an effective way to increase college students’ communication opportunities, enhance the ability of social activities, expand social communication and realize physical fitness and social value. It promotes college students’ self-cultivation and overall quality development and paves the way for students to enter society in the future (Anderlucci et al., 2020). Then, a two-round questionnaire survey is conducted on 90 students before and after particular treatments. During the experiment, the experimental group receives DMT intervention for one semester, the control group takes a sports dance course, and the benchmark group takes no intervention. Meanwhile, these three groups are pre-tested and post-tested with ICQ-CS and PANAS for psychological measurement. The reliability, validity, and applicability of the scale are verified. The internal consistency coefficient of ICQ-CS is between 0.5696 and 0.8217, indicating good reliability; the internal consistency coefficient of PANAS is 0.639 ∼ 0.905, demonstrating excellent reliability. Moreover, Confirmatory Factor Analysis shows that the structural validity of the scale is good (χ^2^/df = 2.60, RM SEA = 0.07, and CFI = 0.96), and the significance level α is 0.05. Therefore, the two scales are suitable for the college students of Xi’an Institute of Physical Education.

The ICQ-CS scale has 25 questions involving five dimensions: interpersonal relationship building ability, moderate rejection ability, self-disclosure ability, conflict-control ability, and emotional supportability. This scale uses the 5-point scoring method. The higher the score, the better the subject’s interpersonal skills. The PANAS scale consists of 20 adjectives describing different emotions and feelings. Items 1, 3, 5, 9, 10, 12, 14, 16, 17, and 19 evaluate positive emotions, and the rest evaluate negative emotions. After each adjective, there are five options, namely definitely not, almost not, maybe, a little, and extremely, scored 1, 2, 3, 4, and 5, respectively. Ultimately, the scores of positive emotions and negative emotions are summed up. A high positive emotional score indicates that an individual is energetic, concentrated, and happy, while a low positive emotional score indicates indifference. A high negative emotional score means an individual’s subjective feeling of confusion and pain, while a low score means calm. A total of 90 questionnaires are distributed. Among them, 30 questionnaires are distributed in the treatment group, with 29 questionnaires recovered; 30 copies are distributed to the control group, with 28 copies recovered; 30 copies are distributed to the benchmark group, with 30 copies recovered. Eighty-seven questionnaires are recovered in total. Excluding an invalid questionnaire from the control group, there are86 valid questionnaires, the recovery rate is 96%, and the effective rate is 98%.

Several preliminary hypotheses are proposed to study the impact of DMT on college students’ positive and negative emotions. Sports dance can stimulate various feelings with the cooperation of the movement and interactions of limbs with different styles of music. Under long-term dance training, dancers will unwittingly apply what they learn in class to real-life and interpersonal communication. Sports dance can accelerate metabolism, improve human function, enhance physical quality, and improve body shape, significantly meeting the aesthetic needs of college students. Hence, sports dance has become one of their trendy lifestyles. DMT emphasizes the importance of physical and mental communication, while sports dance emphasizes the importance of physical exercise; physical exercise and mental health are interrelated.

### Design of the Dance Movement Therapy Intervention

In this section, the three groups are tested two-rounds before and after the experiment. The subjects’ emotional differences between pre-test and post-test are analyzed to evaluate the intervention experiment. Before the intervention experiment, the respondents are informed that the survey data are only used for research to ensure the objectivity and accuracy of the test results (Patel et al., 2018). Respondents only fill in the student number, not their name. The specific research framework can be divided into three stages: pre-test, experimental intervention, and post-test. Two scales are used in the experiment. The treatment group is given a 16-week DMT course once a week. The course is arranged based on the principles and methods of DMT (Jin et al., 2020). Table 1 displays the complete scheme of the DMT intervention.

**TABLE 1:** Intervention design of Dance Movement Therapy (DMT).

A	B	C	D
1	Acquaintance	Preliminary understanding of others	Introduce yourself with language and body movement; get to know each other for the first time by imitating others’ actions.
2	Trust	Build mutual trust	Experience “you are my eyes.”
3	Ego	Know and accept who you really	Subjects try to explore the body’s inner space by breathing and the external area by stretching their limbs to understand the body’s outline.
4	ID	Know and accept who you are	Improvise to understand and improve body shape, creating and stimulating creativity.
5	Others	Build relationships correctly	Subjects play robot games to feel active and passive emotions; subjects feel being accepted by others through mirror practice.
6	Others	Build relationships correctly	Simulate aircraft flight and learn to avoid and control conflicts; speak your feelings and express yourself; mirror practice to explore your suitable role.
7	Team	Build a sense of teamwork	Group performance to establish a sense of mission and teamwork.
8	Exhibition	Cultivate the ability to realize yourself	Show the results in groups.
9	Communication	Find the right role	Subjects are divided into two groups for simple editing and mining their roles.
10	Coordination	Establish mutual understanding	Use a prop together for the first time to complete a simple action.
11	Praise	Develop positive emotions	Say one of the advantages of your partner and give play to positive emotions.
12	Ventilation	Reasonably vent negative emotions	Present negative emotions with language and body.
13	Meditation	Relieve and release pressure	Combine with music therapy.
14	Impromptu	Tap your physical potential	Improvised dance with the music of different emotions.
15	Exhibition	Strengthen interpersonal relationships	Team creative presentation to consolidate interpersonal relationships.
16	Say goodbye	Deepen each other’s feelings	Review the days of ordinary experience, talk about inner feelings and deepen each other’s feelings.

*(A, intervention times; B, intervention theme; C, intervention goal; D, intervention subject) SPSS 25.0 is used for mathematical statistics. Analysis of Variance tests the treatment group, control group, and benchmark group. Paired sample t-test is used to compare the pre-test and post-test results of the three groups. The interview contains a classroom recording and an after-class interview. The original feelings of each student in the treatment group are recorded to evaluate the DMT effect. The interview outline is set, and five students from the treatment group are chosen for a structured interview after completing all courses.*

### Dance Movement Therapy

The 16-week DMT intervention is locally called a creative dance course, which maintains the principles and methods of DMT and meets the psychological needs of Chinese people, ensuring the smooth progress of the intervention. Each intervention lasts 90 min, including 20 min of warm-up, 50 min of main intervention content, and 20 min of relaxation and discussion. Modern dance is one of the three foundations of DMT; it breaks through the stylized training mode of traditional dance and truly expresses life and self, which is consistent with the concept of DMT. Therefore, warm-up activities usually adopt the essential elements and training methods of modern dance, ensuring the relaxation of the body and mood before intervention. The DMT intervention uses the following approaches.

**FIGURE 1:**
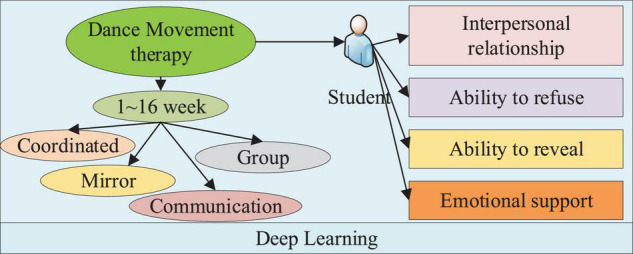
Design of the psychological research on college students under Dance Movement Therapy (DMT).

(1) Coordinated synchronous movement method: dance therapists use the synchronous method to establish emotional acceptance, mutual concern, and mutual trust with students.

(2) Mirror reflection action method: synchronously imitated actions can help students establish self-awareness and deepen communication.

(3) Communication and interactive action method can help students strengthen their physical expression ability with the experience of subjective effort-action shaping.

(4) Application of group dynamics can reduce members’ communication anxiety, remove communication barriers, and improve interpersonal skills through group dynamics experience. Figure 1 shows the specific design concept of DMT for college students.

As shown in Figure 1, various psychological conditions of college students are analyzed through DL-based DMT. It can reflect the specific psychological performance of college students to comprehensively study the particular results of DMT on college students’ psychological activities. The 16-week intervention plan is described in detail below.

During the first 4 weeks, subjects are encouraged to introduce themselves with verbal and body language: the first lesson lets everyone get acquainted. All students, teachers, and teaching assistants stand in a circle. The dance therapist gives instructions. Each person provides a self-introduction by demonstrating a self-invented name-representing body movement, using either a real name or nickname. During this stage, other students imitate their actions until the end of the first round. In the second round, students are encouraged to replicate the steps under a given melody and make the actions consistent with the rhythm and emotion of the music. In this training, individuals might choose their habitual actions to give meaning to their names to let others know them better. Meanwhile, it is believed that their counterparts’ imitation and acceptance of their movements can bring a sense of psychological identity to each individual.

During 5–8 weeks, subjects are encouraged to discover body space. The dance therapist help students find the inner area of their body through breathing practice. This practice is inspired by the idea of breathing meditation in Yoga. Limb extension can guide students to explore the external space of the body. The dance therapist gives three pieces of music with different styles. The students dance freely according to the music, without specified dancing styles or any quality requirements for limb movements. Such impromptu exercises can help students open their bodies, develop their body space, and convey their body feelings to their hearts, thereby releasing pressure and expressing their emotions.

During 9–12 weeks, mirror practice is used, namely “you are my mirror.” Two students stand face to face and dance to the music. One student acts as a prototype, and the other presents his mirror image, that is, active mirror behavior. First, the dance therapist and the teaching assistant demonstrate the mirror practice. Then, students are encouraged to practice in groups. At the end of a piece of music, the partners change their roles. Afterward, simulated aircraft practice is carried out. Students are encouraged to imagine themselves as aircraft to open their arms (wings) and imagine the flight mode and route. Meanwhile, every student is surrounded by many counterparts (airplane), so they should timely change their flight height and angle to adjust the flight route to avoid collision with other aircraft (counterpart), thus completing a safe flight. This exercise is supposed to practice students’ sensitivity and conflict control ability.

During 13–16 weeks, the work is completed with props. All students are divided into three groups, and a students’ home is created with the existing props in the dance room. Props are an essential part of dance choreography. Clever use of props can bring surprisingly favorable effects. In the group activities, each group member works together and actively cooperates, which greatly stimulates the sense of teamwork. The students’ feelings are further deepened in jointly creating their own home. Secondly, subjects are encouraged to use their limb movements to express their emotions: the whole class forms a circle; everyone describes their recent negative emotions using one sentence and denotes the sentiment using a self-invented body movement; when one student does so, the rest imitates. Thereby, the students can safely vent out their destructive emotions under the simultaneous interaction and expression of verbal and body language. By the end of the practice, bad feelings might get away with the body expressions and oral communication.

## Results and Discussion

### Analysis of Pre-test Data of Scale

**FIGURE 2:**
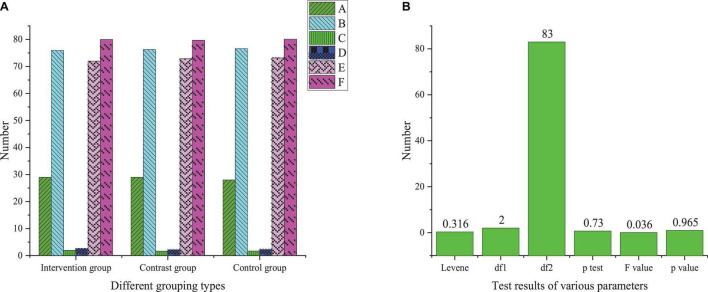
Statistics of pre-test results of interpersonal skills of the treatment group, control group, and benchmark group (A, number of subjects; B, mean; C, standard deviation; D, relative deviation; E, lower limit of 95% confidence interval; F, upper limit of 95% confidence interval; **A** basic information of pre-test; **B** analysis of variance of pre-test results).

Before the intervention of DMT, the interpersonal communication ability of each group is measured, and there is no significant difference in each group. The results are shown in Figure 2.

Figure 2 indicates that the variance of each group meets the quality standard (*P* > 0.05). There is no significant difference in the scores of each group before the test (*F* = 0.036; *P* > 0.05). It suggests that the grouping of experimental objects is reasonable and does not affect the experimental results. Therefore, this paper conducts group intervention on students through DMT and dance courses and comprehensively analyzes the specific intervention methods suitable for college students’ psychological improvement by comparing intervention conditions. Firstly, different research objects are grouped to study the comprehensive impact of diverse interventions on students’ psychology to improve the practicability of this study. Secondly, the psychological status of college students is comprehensively checked through various interventions to deeply analyze the changes of college students and improve the practicability of DMT.

### Investigation and Characteristics of College Students’ Emotional Psychology

**FIGURE 3:**
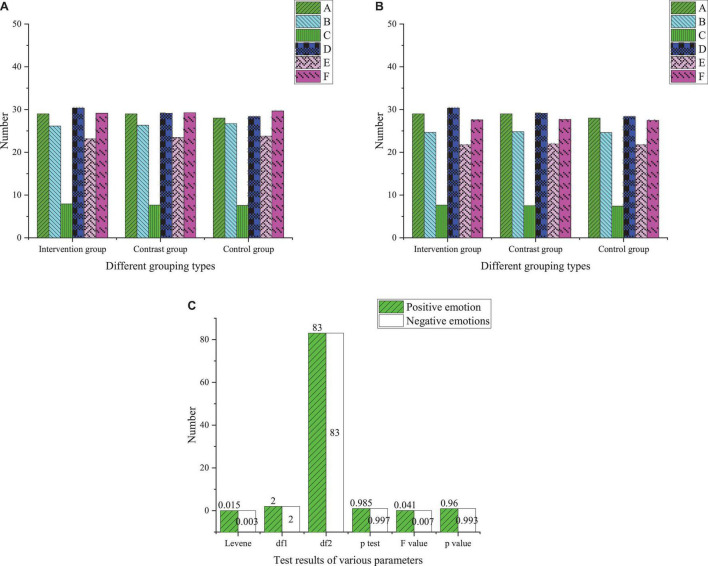
Statistical results of pre-test of emotions of the treatment group, control group, and benchmark group (**A** basic pre-test of positive emotions; **B** basic pre-test of negative emotions; **C** analysis of variance of pre-test of positive and negative emotions; A, number of experiments; B, average; C, standard deviation; D, relative deviation; E, lower limit of 95% confidence interval; F, upper limit of 95% confidence interval).

Before the intervention, research objects are investigated and measured with PANAS to understand the characteristics and possible problems of contemporary college students’ interpersonal communication to provide a basis for designing the DMT intervention course. Before the DMT intervention, each group’s emotional and psychological measurement is carried out, and there is no significant difference in the pre-test, as presented in Figure 3.

According to Figure 3, the pre-test statistical results show that the variance of each group meets the qualitative standard (*P* > 0.05). In addition, there is no significant difference in the pre-test scores among the three groups. Therefore, the experimental objects are grouped reasonably to avoid the impact on the experimental results and ensure the smooth implementation of the follow-up process. Analyzing the pre-test results can provide an analytical benchmark for the psychological changes of college students in the follow-up research and a standard for the research grouping of college students. Therefore, analyzing the psychological status of college students through the pre-test results can get the fundamental psychological changes of students and reduce the error of research grouping of college students.

### Investigation and Characteristics of College Students’ Emotional Psychology

**FIGURE 4:**
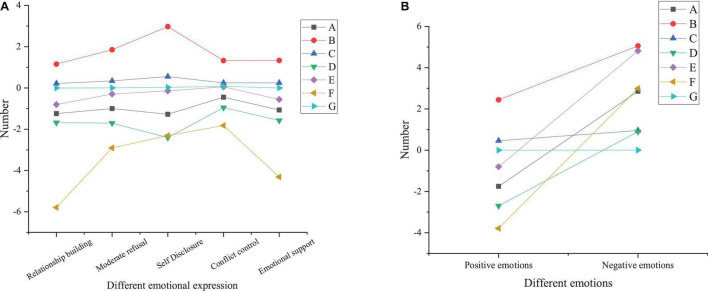
Paired sample *t*-test results of interpersonal relationships of the treatment group before and after the DMT intervention (A, mean; B, standard deviation; C, distribution of sample statistics; D, lower limit of 95% confidence interval; E, upper limit of 95% confidence interval; F, *t*-test; G, *p*-value; **A** analysis of influencing factors; **B** sample *t*-test results).

Figure 4 reveals the paired sample *t*-test results of interpersonal relationships of the treatment group before and after the DMT intervention.

**FIGURE 5:**
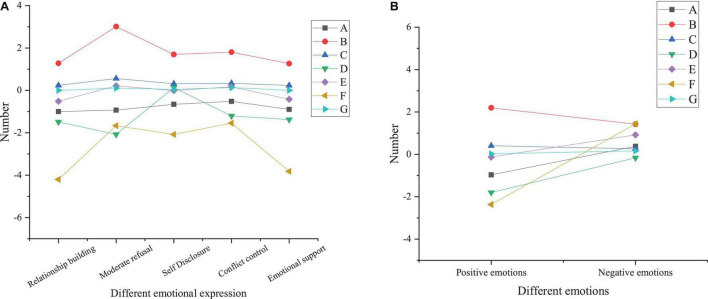
Paired sample *t*-test results of the pre-test and post-test of the control group (A, mean; B, standard deviation; C, distribution of sample statistics; D, lower limit of 95% confidence interval; E, upper limit of 95% confidence interval; F, *t*-test; G, *p*-value; **A** analysis of influencing factors; **B** sample *t*-test results).

From Figure 4, the treatment group shows no significant difference in conflict control ability before and after the intervention (*p* = 0.079 > 0.05) while demonstrating substantial differences in other aspects (*p* < 0.05). After the DMT intervention, the total score of interpersonal relationships and the score of interpersonal relationship-building ability, moderate rejection ability, self-disclosure ability, and emotional supportability of the treatment group significantly increases. This indicates that the DMT intervention has a significant effect on improving college students’ interpersonal relationships. The reason may be that the whole 16-week DMT program is as per the concept of interpersonal communication. The intervention content emphasizes self-awareness, self-acceptance, and acceptance of others. For example, practice like “introducing yourself with language and body movement” can help subjects get to know each other while bringing a sense of psychological identity when their actions are accepted and imitated; under “Mirror practice,” students will have different physical and mental feelings when they partner with different people, which is helpful for students to understand each other; the practice of “group presentation” can cultivate the sense of teamwork, leadership, and cooperation, which also need one another’s emotional support and conflict control ability. In summary, DMT plays a positive moderating role in college students’ interpersonal relationships, which is suitable for psychological education courses in higher institutions. Figure 5 demonstrates the paired sample t-test results of the pre-test and post-test of the control group.

**FIGURE 6:**
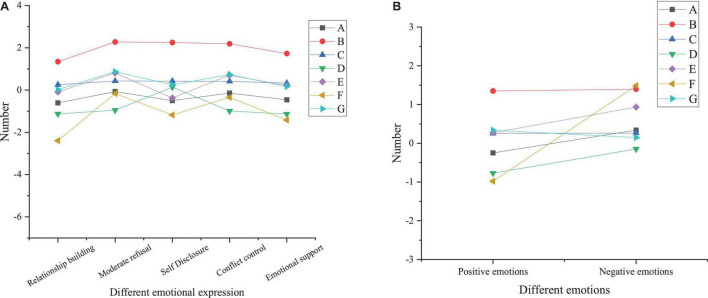
Paired sample *t*-test results of the pre-test and post-test of the benchmark group (A, mean; B, standard deviation; C, distribution of sample statistics; D, lower limit of 95% confidence interval; E, upper limit of 95% confidence interval; F, *t*-test; G, *p*-value; **A** analysis of influencing factors; **B** sample *t*-test results).

Through Figure 5A, the control group shows differences in the total score of interpersonal relationships and the score of interpersonal relationship-building ability, moderate rejection ability, self-disclosure ability, and emotional supportability before and after the sports dance course (*p* < 0.05), with no significant difference in the intermediate rejection ability and conflict control ability (*p* > 0.05). Figure 5B suggests that there is a difference in positive emotions of the control group before and after the course (*p* = 0.025 < 0.05); there is no significant difference in negative emotions (*p* = 0.163 > 0.05). It proves that sports dance has a specific effect on stimulating students’ positive emotions, but it has no significant impact on improving negative emotions. Figure 6 illustrates paired sample *t*-test results of the pre-test and post-test of the benchmark group.

Figure 6A shows that there is no significant difference in the total score of interpersonal relationships and the score of the moderate rejection ability, self-disclosure ability, conflict control ability, and emotional supportability of the benchmark group before and after the experiment (*p* > 0.05), but there is the difference in the relationship-building ability (*p* < 0.05). The reason is that the pre-test is at the beginning of the school semester, and the post-test is at the end of the semester; after a semester of communication, students are likely to understand each other. Therefore, the benchmark group has differences in the relationship-building ability before and after the experiment, and, consequently, the post-test score is greater than the pre-test score. Figure 6B proves that there is no significant difference in positive and negative emotions of the benchmark group between the pre-test and post-test (*p* > 0.05), which is consistent with the above conclusions.

Overall, the above experimental results indicate that among the three experimental groups, the conflict control ability of the treatment group is the same as that of other groups, and there is no significant difference before and after treatment. Still, there is a significant difference compared with the control group and the benchmark group in other aspects, and the ability of the treatment group is significantly improved in these aspects. Still, there is no difference between the benchmark and control groups. Therefore, it can be concluded that DMT has apparent advantages in the psychotherapy of college students, can significantly treat the psychological problems of college students, and help students learn better.

### Analysis of the Effect of Dance Movement Therapy on College Students’ Psychological Education

Through the DMT intervention experiment on the experimental subjects, after the intervention, the total score of the IMS-CS, and scores of the interpersonal relationship building ability, moderate rejection ability, self-disclosure ability, conflict control ability, and emotional support-ability of experimental subjects have been significantly improved. It shows that the DMT intervention has a noticeable effect on improving college students’ interpersonal relationships. This is because, in the process of DMT, the DMT is designed to implement the concept of interpersonal communication. The therapy activities emphasize knowing oneself, knowing others, accepting oneself, and accepting others. For example, through the self-introduction of language and body movements, students can understand each other and produce psychological identity when their behavior is accepted and imitated because mirror practice can improve students’ mutual understanding. Then, the emotional impact of the DMT intervention experiment on subjects is analyzed. After the sports dance classes, the control group shows differences in interpersonal relationships, relationship-building ability, self-disclosure ability, and emotional supportability, but no significant difference in moderate rejection and conflict-control abilities. It indicates that DMT has a better effect on stimulating students’ positive emotions than traditional treatment methods. Therefore, the DMT scheme reported here is highly effective for college mental health treatment.

Compared with the traditional dance courses (Koch et al., 2019; Chao et al., 2021), the open curriculum formally turns the dry knowledge into games and improvisation by integrating the comprehensive understanding of mechanics, aesthetics, and psychology. Utilizing props, music, poetry, and art, students can more deeply understand the professional theoretical knowledge in dance creation and practice, such as time, space, motivation, force effect, and scheduling. In addition, the open curriculum emphasizes students’ self-expression, especially the transference of inner feelings through dance. In terms of content (Zan et al., 2021), the available curriculum pays more attention to group learning and practices, often involving multiple people, even groups simultaneously. Through communication and cooperation, students can continue to learn in new ways. Teachers can timely understand and solve the problems catering to students’ learning and practicing needs, hence promoting students’ comprehensive development with rich course content encompassing various dance arts, such as classical dance, national folk dance, modern dance, contemporary dance, ballet, and jazz dance. Meanwhile, there is no restriction on the form of expression. Diversified types of dances, such as solo dance, duet dance, trio dance, and group dance, can be encouraged. Students can create according to their interests and learn about the relationship between man, society, nature, and dance. Furthermore, students learn how to get along with others and the external world in cooperation and communication. They can strengthen their excellent characters and learn from others. The dance itself is a way to enhance the body. Dance creation and practice courses help dancers express their true feelings through dance movements and guide students to respect others’ opinions or inner thoughts through collaborative practice. The effect of dance might go beyond simple body movements to enable students to understand their relationship with the world, society, and other multi-level relations through their sensory system. Finally, through the integration with multiple disciplines, the open curriculum plays a critical role in cultivating students’ physical quality, spatial consciousness, and aesthetic cultivation and promotes the comprehensive development of students.

### Discussion

This paper studies the application of DMT in college students’ LDE based on educational psychology. Overall, this paper sets up three experimental groups and analyzes the rationality of grouping before the experiment. The empirical findings corroborate that DMT plays a significant positive role in LDE college students. Table 2 specifies the data comparison under DMT.

**TABLE 2:** Comparison of the three groups.

Component	Factor	Before intervention	After intervention (*p*-value)
Treatment group	Control ability	*F* = 0.036 *p* > 0.05	>0.05
	Others		<0.05
	Positive emotions		<0.05
	Negative emotions		<0.05
Control group	Moderate rejection and Conflict control		>0.05
	Others		<0.05
	Positive emotions		<0.05
	Negative emotions		>0.05
Benchmark group	Build relationships		<0.05
	Others		>0.05
	Positive emotions		>0.05
	Negative emotions		>0.05

As shown in Table 2, after DMT intervention, all abilities except for conflict control of this group have changed significantly, both in positive and negative emotions. Besides, the control group has no significant changes in all abilities except for moderate rejection and conflict control and has substantial positive emotional shifts. In contrast, there is no significant change in other skills of the benchmark group except for establishing relationships. Therefore, DMT has a significant positive impact on college students’ LDE and can help college students cultivate comprehensive quality. Compared with the research of Yao and Zhang (2021), the grouping of research objects in this paper is more reasonable, and the research content is more prosperous. As a result, the research results are more affordable and provide more accurate reference content for college students’ psychological education.

## Conclusion

Based on the LDE of college students, the immediate and long-term effects of DMT on college students’ introspection ability, self-awareness, and emotion regulation are studied from the perspective of educational psychology. The influencing factors and substantive benefits of DMT are discussed. The results show a significant difference in the pre-test among the treatment, control, and benchmark groups (*p* > 0.05). Besides, the treatment group shows no significant change in the conflict control ability before and after the DMT intervention (*p* = 0.079 > 0.05), but with substantial differences in other aspects (*p* < 0.05); meanwhile, after the intervention, the total score of interpersonal relationships and the scores of the interpersonal relationship building ability, moderate refusal ability, self-disclosure ability, and emotional supportability grow dramatically. Moreover, the control group presents improvements in interpersonal relationships, interpersonal relationship building ability, self-disclosure ability, and emotional supportability after the sports dance course (*p* < 0.05), but there is no significant difference in the moderate refusal ability and conflict control ability (*p* > 0.05). For the benchmark group, there is no significant difference in the total score of interpersonal relationships and the scores of moderate rejection ability, self-disclosure ability, conflict control ability, and emotional supportability before and after the experiment (*p* > 0.05), but there is a significant difference in the relationship-building ability (*p* < 0.05). Furthermore, there are prominent differences in the treatment group’s positive emotions and negative emotions before and after the intervention (*p* < 0.05); the positive emotion score has increased significantly, while the negative emotion score has decreased dramatically. There is a difference in positive emotions of the control group before and after the sports dance course (*p* = 0.025 < 0.05). Still, there is no significant difference in negative emotions before and after the course (*p* = 0.163 > 0.05). There is also no significant difference in positive and negative emotions of the benchmark group before and after the test (*p* > 0.05). Nevertheless, some deficiencies have been found. For example, this experiment did not take sufficient and comprehensive information, such as specialty and family backgrounds. Besides, the effect of DMT on students is only tested through respondents majoring in dance. Follow-up research will extend the sample scale and involve students in other majors for more comprehensive and persuasive results.

## Data Availability Statement

The raw data supporting the conclusions of this article will be made available by the authors, without undue reservation.

## Ethics Statement

The studies involving human participants were reviewed and approved by Hunan University of Arts and Sciences Ethics Committee. The patients/participants provided their written informed consent to participate in this study. Written informed consent was obtained from the individual(s) for the publication of any potentially identifiable images or data included in this article.

## Author Contributions

Both authors listed have made a substantial, direct, and intellectual contribution to the work, and approved it for publication.

## Conflict of Interest

The authors declare that the research was conducted in the absence of any commercial or financial relationships that could be construed as a potential conflict of interest.

## Publisher’s Note

All claims expressed in this article are solely those of the authors and do not necessarily represent those of their affiliated organizations, or those of the publisher, the editors and the reviewers. Any product that may be evaluated in this article, or claim that may be made by its manufacturer, is not guaranteed or endorsed by the publisher.

## References

[B1] Acharya L. Jin L. Collins W. (2018). College life is stressful today–Emerging stressors and depressive symptoms in college students. *J. Am. Coll. Health* 66 655–664. 10.1080/07448481.2018.1451869 29565759

[B2] Alexander B. Florian H. Peter Z. Laux G. (2021). Driving performance under treatment of most frequently prescribed drugs for mental disorders: a systematic review of patient studies. *Int. J. Neuropsychopharmacol.* 21 679–693. 10.1093/ijnp/pyab031 34038545 PMC8453274

[B3] Al-Zoubi E. M. (2020). Quality of life and its relationship with cognitive flexibility among higher education students. *J. Educ. Soc. Res.* 10:156. 10.36941/jesr-2020-0075

[B4] Anderlucci L. Lubisco A. Mignani S. (2020). Investigating the judges performance in a national competition of sports dance. *Soc. Indic. Res.* 45:11.

[B5] Biondo J. Gerber N. (2020). Single-session dance/movement therapy for people with acute schizophrenia: development of a treatment protocol. *Am. J. Dance Ther.* 42 277–295.

[B6] Borg M. E. Butterfield K. M. Wood E. Zhang H. H. Pinto S. (2021). Investigating the impacts of personality on the use and perceptions of online collaborative platforms in higher education. *SN Soc. Sci.* 1:40. 10.1007/s43545-020-00053-x 34693305 PMC7847746

[B7] Campbell B. (2019). Past, present, future: a program development project exploring post-traumatic slave syndrome (PTSS) using experiential education and dance/movement therapy informed approaches. *Am. J. Dance Ther.* 41 214–233.

[B8] Chao H. W. Wu C. C. Tsai C. W. (2021). Do socio-cultural differences matter? A study of the learning effects and satisfaction with physical activity from digital learning assimilated into a university dance course. *Comput. Educ.* 165:104150.

[B9] Cook T. Roy A. R. K. Welker K. M. (2019). Music as an emotion regulation strategy: an examination of genres of music and their roles in emotion regulation. *Psychol. Music* 47 144–154.

[B10] Cramer C. (2018). Belastung und beanspruchung im lehrerinnen-und lehrerberuf: übersicht zu theorien, variablen und ergebnissen in einem integrativen rahmenmodell. *bildungsforschung* 1, 1–23. 10.25656/01:16575

[B11] Deng X. Guo X. Wu Y. J. Chen M. (2021). Perceived environmental dynamism promotes entrepreneurial team member’s innovation: explanations based on the uncertainty reduction theory. *Int. J. Environ. Res. Public Health* 18:2033. 10.3390/ijerph18042033 33669732 PMC7921965

[B12] Dryman M. T. Heimberg R. G. (2018). Emotion regulation in social anxiety and depression: a systematic review of expressive suppression and cognitive reappraisal. *Clin. Psychol. Rev.* 65 17–42. 10.1016/j.cpr.2018.07.004 30064053

[B13] Ekberg D. Danby F. Rendle-Short J. Herbert A. Bradford N. K. Yates P. (2019). Discussing death: making end of life implicit or explicit in paediatric palliative care consultations. *Patient Educ. Couns.* 102 198–206. 10.1016/j.pec.2018.08.014 30236971

[B14] Feng B. Sun K. Zhong Z. Chen M. (2021). The internal connection analysis of information sharing and investment performance in the venture capital network community. *Int. J. Environ. Res. Public Health* 18:11943. 10.3390/ijerph182211943 34831699 PMC8624762

[B15] Fox J. Vendemia M. A. Smith M. A. Brehm N. R. (2021). Effects of taking selfies on women’s self-objectification, mood, self-esteem, and social aggression toward female peers. *Body Image* 36 193–200. 10.1016/j.bodyim.2020.11.011 33360476

[B16] Goodill S. W. (2021). Chaiklin, S. & Wengrower, H. (Eds.) (2021) dance and creativity within dance/movement therapy: international perspectives. Routledge. *Am. J. Dance Ther.* 43 1–5.

[B17] Grasser L. R. Al-Saghir H. Wanna C. Spinei J. Javanbakht A. (2019). Moving through the trauma: dance/movement therapy as a somatic-based intervention for addressing trauma and stress among syrian refugee children. *J. Am. Acad. Child. Adolesc. Psychiatry* 58 1124–1126. 10.1016/j.jaac.2019.07.007 31348987

[B18] Jin S. Vegelius J. Wallentin Y. (2020). A marginal maximum likelihood approach for extended quadratic structural equation modeling with ordinal data. *Struct. Equ. Model.* 27 864–873.

[B19] Jordan M. (2018). Conditional adversarial domain adaptation. *Adv. Neural. Inf. Process. Syst.* 31, 12–16. 10.1007/978-3-030-01237-3_9

[B20] Khan I. A. (2020). Dance movement therapy: a promising lifestyle intervention in the management of chronic obstructive pulmonary disease. *J. Integr. Tradit. Chin. Western Med.* 8:6.

[B21] Koch S. C. Riege R. Tisborn K. Biondo J. Martin L. Beelmann A. (2019). Effects of dance movement therapy and dance on health-related psychological outcomes. a meta-analysis update. *Front. Psychol.* 10:1806. 10.3389/fpsyg.2019.01806 31481910 PMC6710484

[B22] Kümpfen F. Maurer J. (2018). Does education help “old dogs” learn “new tricks”? The lasting impact of early-life education on technology use among older adults. *Res. Policy* 47 1125–1132.

[B23] Li W. Dorstyn D. S. Jarmon E. (2020). Identifying suicide risk among college students: a systematic review. *Death Stud.* 44 450–458. 10.1080/07481187.2019.1578305 30836043

[B24] Liu C. H. Stevens C. Wong S. H. M. Yasui M. Chen J. A. (2019). The prevalence and predictors of psychological health diagnoses and suicide among US college students: implications for addressing disparities in service use. *Depress. Anxiety* 36 8–17. 10.1002/da.22830 30188598 PMC6628691

[B25] Malin E. Pedersen N. L. Johansson A. Fors S. Dahl Aslan A. K. (2020). Life-course socioeconomic differences and social mobility in preventable and non-preventable mortality: a study of Swedish twins. *Int. J. Epidemiol.* 48 1701–1709. 10.1093/ije/dyz042 30929008 PMC6857748

[B26] Michels K. Dubaz O. Hornthal E. Bega D. (2018). “Dance therapy” as a psychotherapeutic movement intervention in Parkinson’s disease. *Complement. Ther. Med.* 40 248–252. 10.1016/j.ctim.2018.07.005 30219460

[B27] Oliynyk N. M. Kravets N. Y. Pokryshko O. V. Yelagina N. (2020). The role of higher education in the formation of students’ healthy lifestyle. *Медична освіта* 2 96–102.

[B28] Patel D. K. (2020). Building emotional resilience and physical stability through movement therapy for sports (MTS^®^): a case for applying MTS^®^ as a tool for enhancing athletic performance among professional or trained athletes. *Am. J. Dance Ther.* 42 256–276.

[B29] Patel N. K. Nivethitha L. Mooventhan A. (2018). Effect of a yoga-based meditation technique on emotional regulation, self-compassion, and mindfulness in college students. *Explore* 14 443–447. 10.1016/j.explore.2018.06.008 30366832

[B30] Phan H. P. Bing H. N. Chen S. C. Wu L. Shih J. H. Shi S. Y. (2021). Life, death, and spirituality: a conceptual analysis for educational research development. *Heliyon* 7:e06971. 10.1016/j.heliyon.2021.e06971 34036188 PMC8138599

[B31] Prieto L. R. (2020). Concerns about teaching culturally diverse students in a cross-disciplinary sample of higher education faculty. *Teach. High. Educ.* 34, 1–12. 10.1080/13562517.2020.1863346

[B32] Rogoza R. Żemojtel-Piotrowska M. Kwiatkowska M. M. Kwiatkowska K. (2018). The bright, the dark, and the blue face of narcissism: the Spectrum of Narcissism in its relations to the meta traits of personality, self-esteem, and the nomological network of shyness, loneliness, and empathy. *Front. Psychol.* 9:343. 10.3389/fpsyg.2018.00343 29593627 PMC5861199

[B33] Rogti M. (2020). Contribution of meta-cognitive competence in preserving student self-regulation in Algerian higher education: a case from ENS of laghouat. *Arab World Engl. J.* 11 445–460.

[B34] Shin J. Kim M. S. Hwang H. Lee B. (2018). Effects of intrinsic motivation and informative feedback in service-learning on the development of college students’ life purpose. *J. Moral Educ.* 47 159–174. 10.1080/03057240.2017.1419943

[B35] Sousa D. Pestana A. Tavares A. (2019). Self-awareness, verbalization and new meanings as the heart and soul of significant events in existential psychotherapy. *J. Contemp. Psychother.* 49 161–167. 10.1007/s10879-018-9410-2

[B36] Suskin G. Al-Yagon M. (2020). Culturally sensitive dance movement therapy for ultra-orthodox women: group protocol targeting bodily and psychological self-perceptions. *Arts Psychother.* 71:101709. 10.1016/j.aip.2020.101709

[B37] Tavormina R. Tavormina M. G. M. (2018). Overcoming depression with dance movement therapy: a case report. *Psychiatr. Danubina* 30(Suppl. 7) 515–520. 30439838

[B38] Testoni I. Biancalani G. Ronconi L. Varani S. (2019). Let’s start with the end: bibliodrama in an Italian death education course on managing fear of death, fantasy-proneness, and alexithymia with a mixed-method analysis. *OMEGA J. Death Dying* 67:003022281986361. 10.1177/0030222819863613 31366309

[B39] Tortora S. (2019). Complementary and alternative medicine creative arts therapy dance/movement psychotherapy dance/movement therapy integrative medicine integrative oncology pediatric cancer symptom management%trauma and PTSD. *Children (Basel, Switzerland)* 6:4. 10.3390/children6010014 30669668 PMC6352017

[B40] Viere T. Amor B. Berger N. Fanous R. D. Arduin R. H. Keller R. (2021). Teaching life cycle assessment in higher education. *Int. J. Life Cycle Assess.* 26 511–527. 10.1007/s11367-020-01844-3 33349738 PMC7744451

[B41] Wang K. (2019). Research on the multi-dimensional ideological and political education of college students based on the computer platform. *Rev. Fac. Ing.* 32 447–454.

[B42] Wang L. Tian Z. (2019). A case study of dancing therapy for an aggressive juvenile. *Sci. Educ. Article Collects* 9 170–185.

[B43] Wang W. Qian G. Wang X. Lei L. Hu Q. Chen J. (2019). Mobile social media use and self-identity among Chinese adolescents: the mediating effect of friendship quality and the moderating role of gender. *Curr. Psychol.* 2 12–13.

[B44] Woodford M. R. Weber G. Nicolazzo Z. Hunt R. Kulick A. Coleman T. (2018). Depression and attempted suicide among LGBTQ college students: fostering resilience to the effects of heterosexism and cisgenderism on campus. *J. Coll. Stud. Dev.* 59 421–438.

[B45] Wyckoff C. Preston J. (2021). Fall: the mysterious life and death of Robert Maxwell, Britain’s most notorious media baron. *Publ. Res. Q.* 37 1–3.

[B46] Yao F. Zhang A. (2021). Integration of education management and mental health in psychological crisis intervention in colleges and universities. *ASP Trans. Psychol. Educ.* 1 31–38.

[B47] Ye S. Chen M. (2021). Leveraging team expertise location awareness in improving team improvisation: a dynamic knowledge integration perspective. *Psychol. Res. Behav. Manag.* 14 2135–2146. 10.2147/PRBM.S341685 34984035 PMC8700443

[B48] Zan G. Zhang T. Stodden D. F. (2021). Children’s physical activity levels and psychological correlates in interactive dance versus aerobic dance. *Sports Health Sci.* 2 146–151.

[B49] Zhang M. Zhang J. Zhang F. Zhang L. Feng D. (2018). Prevalence of psychological distress and the effects of resilience and perceived social support among Chinese college students: does gender make a difference. *Psychiatry Res.* 267 409–413. 10.1016/j.psychres.2018.06.038 29960938

